# Baicalein Inhibits *Streptococcus mutans* Biofilms and Dental Caries-Related Virulence Phenotypes

**DOI:** 10.3390/antibiotics10020215

**Published:** 2021-02-21

**Authors:** Aparna Vijayakumar, Hema Bhagavathi Sarveswari, Sahana Vasudevan, Karthi Shanmugam, Adline Princy Solomon, Prasanna Neelakantan

**Affiliations:** 1 Quorum Sensing Laboratory, Centre for Research in Infectious Diseases (CRID), School of Chemical and Biotechnology, SASTRA Deemed to be University, Thanjavur 613401, India; aparnavkas11@gmail.com (A.V.); hema.b@live.com (H.B.S.); savvysahana@gmail.com (S.V.); karthi.bioinfo@gmail.com (K.S.); 2 Faculty of Dentistry, The University of Hong Kong, Hong Kong

**Keywords:** baicalein, biofilm, dental caries, hydrophobicity, hydroxyflavone, *Streptococcus mutans*

## Abstract

Dental caries, the most common oral disease, is a major public healthcare burden and affects more than three billion people worldwide. The contemporary understanding of the need for a healthy microbiome and the emergence of antimicrobial resistance has resulted in an urgent need to identify compounds that curb the virulence of pathobionts without microbial killing. Through this study, we have demonstrated for the first time that 5,6,7-trihydroxyflavone (Baicalein) significantly downregulates crucial caries-related virulence phenotypes in *Streptococcus mutans*. Baicalein significantly inhibited biofilm formation by *Streptococcus mutans* UA159 (MBIC_50_ = 200 μM), without significant growth inhibition. Notably, these concentrations of baicalein did not affect the commensal *S. gordonii*. Strikingly, baicalein significantly reduced cell surface hydrophobicity, autoaggregation and acid production by *S. mutans*. Mechanistic studies (qRT-PCR) showed downregulation of various genes regulating biofilm formation, surface attachment, quorum sensing, acid production and competence. Finally, we demonstrate the potential translational value of baicalein by reporting synergistic interaction with fluoride against *S. mutans* biofilms.

## 1. Introduction

Dental caries is a microbial-mediated oral disease initiated by the presence of stagnant plaque biofilms, where pathogens such as *S. mutans* metabolise dietary sugars and produce acids [1]. This results in dysbiosis, which sustains *S. mutans* biofilm growth, creating acidic microenvironments that demineralise the dental hard tissues, leading to dental caries [2]. Acid production and the glucan-rich matrix in *S. mutans* biofilms enable the spatial orientation of other cariogenic microbes constituting an elaborate microbial network leading to the development of caries [1,3]. In addition, multiple virulence factors, including acid and stress tolerance, cell persistence, genetic competence and bacteriocin production collectively contribute to the progression of dental caries [4]. 

The currently available options in preventing or treating dental caries predominantly involve the use of fluoride. Fluoride compounds are somewhat effective in inhibiting demineralisation and enhancing remineralisation of the tooth structure. Even though fluoride exhibits bactericidal activity it cannot inhibit biofilm [5]. Furthermore, repeated usage of high concentrations of fluoride has been linked to the development of fluorosis, decrease in bone density and neural toxicity [6,7]. *S. mutans* strains exhibiting fluoride resistance have already been reported [8]. Taken together, the high prevalence of dental caries and the drawbacks of currently used strategies demonstrates the pressing need to identify more effective, efficient, and non-toxic alternative treatment options, that can potentially synergise with fluorides, so that they can be easily incorporated into commercially available oral care products. 

Although caries is a polymicrobial disease, *S. mutans* is considered as the primary driver for the initiation and progression of caries [3,9]. There has been considerable argument on what the disease-drivers and commensal-keepers in caries are, with various groups demonstrating different microbiome profiles in caries in an age-dependent manner [10,11,12]. That said, the concept of “keystone pathogen” is probably only relevant if the designed strategies are microbicidal in nature. However, given the current understanding of the need for a health microbiome, it is important to identify compounds that can suppress the cariogenic virulence determinants instead of affecting the growth and survival of the microbes [13]. Natural products are indeed a safe approach for rapid clinical translation since they are generally recognised as safe by the United States Food and Drug Administration [14].

Baicalein (5,6,7-trihydroxyflavone), a flavonoid isolated from the roots of *Scutellaria baicalensis* and *Scutellaria lateriflora* is known to exhibit a wide range of biological properties [15,16,17,18]. Previous studies have demonstrated that baicalein, both in naked, as well as nanoparticle-encapsulated forms were biocompatible to human gingival epithelial cells [19]. Furthermore, from the systemic context, baicalein has demonstrated hepatoprotective properties [20]. There is evidence to show the anti-quorum sensing activity and biofilm inhibitory potential of Baicalein against *Staphylococcus aureus* [21], Avian Pathogenic *E. coli* (APEC) [22] and *Candida albicans* [23]. Based on this premise, we hypothesised that baicalein inhibits *S. mutans* biofilms and its virulence. The aim of this study was to characterise the effects of baicalein on the growth and biofilm formation of *S. mutans* along with its associated virulence factors. Furthermore, the potential mechanisms of these effects were studied using transcriptomic analysis.

## 2. Results and Discussion

### 2.1. Baicalein Inhibit S. mutans UA159 Biofilm and Its Virulence Potential

**Figure 1:**
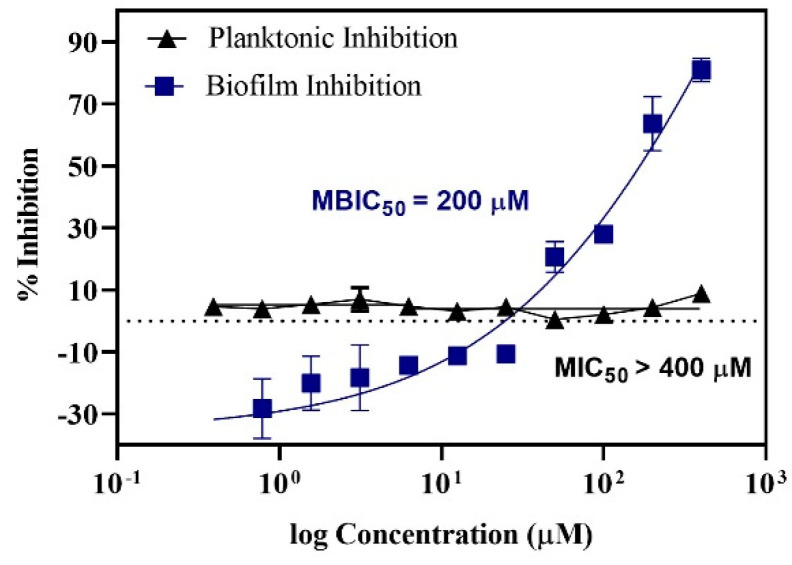
Dose response curve representing the action of Baicalein on *S. mutans*. MBIC_50_ (Minimum Biofilm Inhibitory Concentration) = 200 μM; MIC_50_ (Minimum Inhibitory Concentration) > 400 μM.

**Figure 2:**
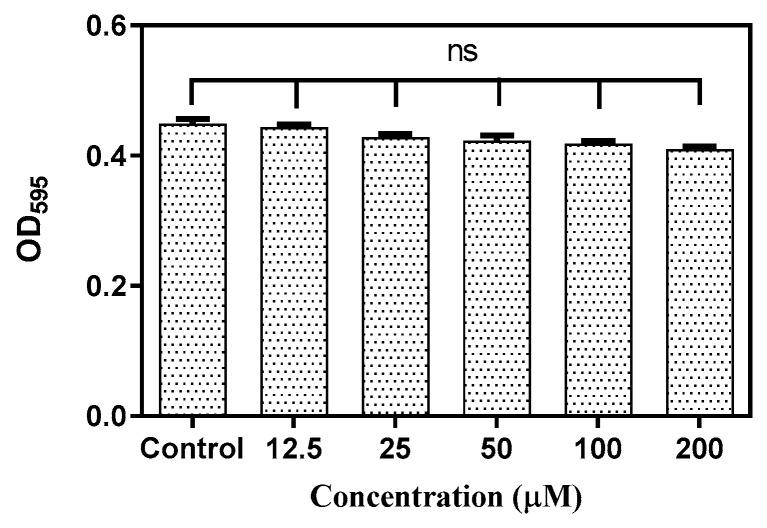
Effect of Baicalein on *S. gordonii* growth at varying concentrations. Student *t* test was used for the significance analysis. *p* < 0.05 was considered significant. ns denotes non-significance.

Figure 1 shows the planktonic and biofilm inhibitory actions of baicalein. There was no significant growth inhibition within the concentration range tested. However, biofilm quantification showed a concentration-dependent inhibition of biofilm with ~50% inhibition at 200 μM and ~80% inhibition at 400 μM with negligible impact on the planktonic growth (<10% inhibition). In addition, the tested concentrations of baicalein had no significant impact on the growth of the commensal *Streptococcus gordonii* (Figure 2). 

**Figure 3:**
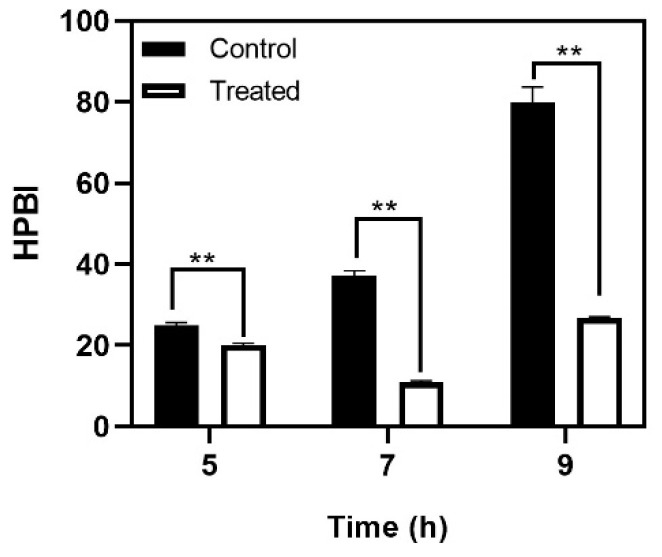
Effect of baicalein on *S. mutans* cell surface hydrophobicity. Student *t*-test was used for analysing significance and *p* < 0.05 was considered significant. ** denotes *p* ≤ 0.05.

*Streptococcus mutans* is known to be one of the pioneers of tooth surface colonization and is often considered the model organism for studying novel therapies. The biofilm matrix production in *S. mutans* is enhanced in the presence of a sucrose rich environment. However, bacterial cell-to-substrate interactions can also take place in a sucrose-independent manner, mediated by hydrophobic interactions [24]. Cell surface hydrophobicity is an important determinant of bacterial surface attachment. Non-hydrophobic cells (those with a low hydrophobicity score) display very weak surface adhesion, and consequently poor biofilm formation. Baicalein decreased the cell surface hydrophobicity at the 5th and 7th hour of treatment (Figure 3). At the 9th hour, a significant decrease in the hydrophobicity was observed wherein the hydrophobicity index was 25%, compared to the untreated control (hydrophobicity index >70%). 

**Figure 4:**
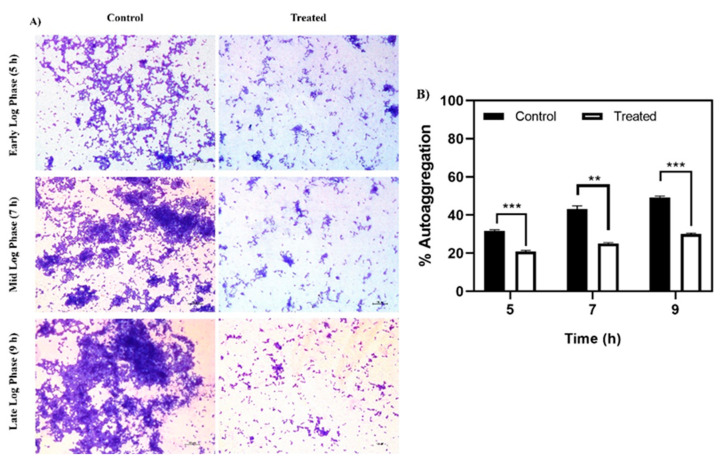
Effect of baicalein on autoaggregation. (**A**) Microscopic analysis of the reduction in autoaggregation for the baicalein treated cells in time dependent manner when compared to control. (**B**) Reduction in % autoaggregation. Student *t*-test was used for analysing significance and *p* < 0.05 was considered significant. * denotes *p* ≤ 0.05, ** denotes *p* ≤ 0.01 and *** denotes *p* ≤ 0.001.

Autoaggregation, a phenomenon where bacterial cells of the same species secrete specific polymers to interact and aggregate, is enhanced by hydrophobic interactions amongst cells [25], and is considered an initial step in biofilm formation. Baicalein treatment showed a marked reduction in autoaggregation at all the three time points: Early log phase (5th hour), mid-log phase (7th hour) and late log phase (9th hour) (Figure 4). The differences were more distinct in the late log phase where baicalein treatment reduced the aggregation by at least 20% relative to the control. Light microscopic images clearly demonstrated the inhibition of autoaggregation by baicalein at different time points considered (Figure 4A).

**Figure 5:**
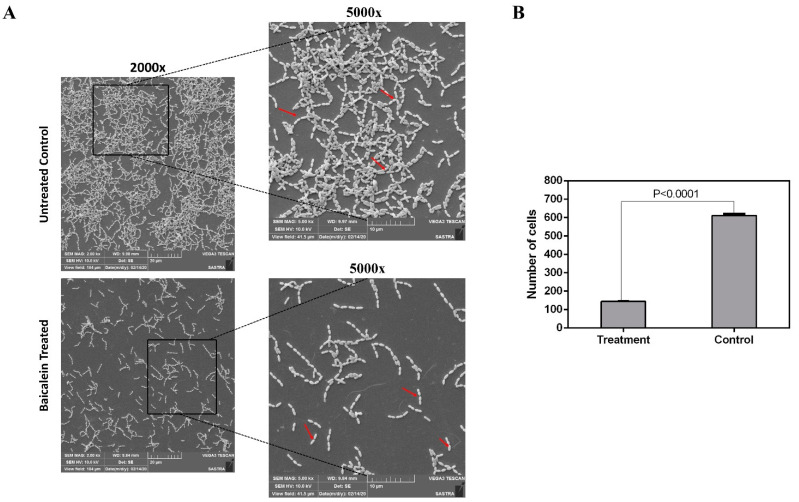
(**A**) SEM (Scanning Electron Microscopy) micrograph depicting the effect of baicalein on biofilm formation. Arrows indicate the long and short chains of *S. mutans* in control and treatment, respectively. (**B**) Reduction in number of cells in the baicalein treated when compared to untreated control biofilms where the treated cells displayed an adhered cell count of <100 cells when compared to the untreated control which had around >500 adhered cells.

The SEM analysis was concurrent with the findings of the cell surface hydrophobicity and autoaggregation assays, and the changes observed in topography, disruption in biofilm architecture, decreased adhered cell count and reduction in cell clustering can be attributed to the ability of baicalein to affect these factors negatively. SEM analysis indicated a decrease in *S. mutans* biofilm biomass when treated with 200 μM of baicalein. A significant reduction in bacterial cell chain length and a 5-fold decrease in adhered cell number was observed in the baicalein treated biofilms (Figure 5). Therefore, baicalein impedes the ability of *S. mutans* to successfully colonize the surface, thereby reducing initial biofilm formation. This initial biofilm serves as a potential adhesion site for other pathogenic oral bacteria in a polymicrobial environment [1]. These findings highlight the possibility of using baicalein as an antifouling coating on substrates. 

**Figure 6:**
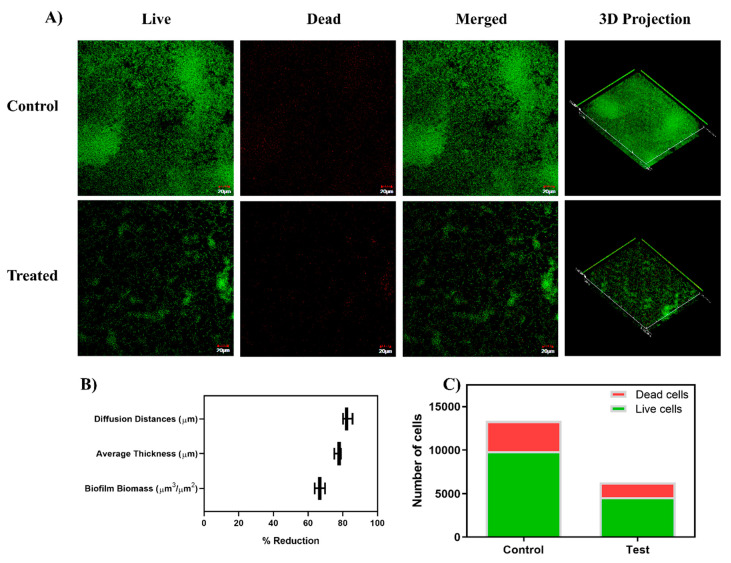
(**A**) CLSM (Confocal Laser Scanning Microscopy) micrographs illustrating the effect on the sub-inhibitory concentration of baicalein (200 µM) on biofilm architecture and viability of the biofilm cells through Live/Dead staining. Baicalein treated cell cultures show sparing green color cells with no confluent mass, indicating inhibition of biofilm formation. (**B**) COMSTAT analysis of the CLSM images revealed a reduction in the biofilm biomass, average thickness, and diffusion distances in the baicalein treated biofilms compared to the untreated control. (**C**) ImageJ analysis shows the proportion of viable cells in the biofilm formed with baicalein treatment at the concentration of 200 µM (MBIC_50_).

The CLSM analysis further strengthened the experimental evidence on biofilm inhibitory effect of baicalein (Figure 6). The architectural analysis of the untreated biofilm and baicalein treated biofilm showed ~70% reduction in the biofilm biomass and ~ 75% reduction in the average thickness of the biofilm matrix. The ~80% reduction of the diffusion distance in the baicalein treated biofilm when compared to the untreated control, confirms the reduced solute movement in the biofilm [26]. These results corroborate the biofilm inhibitory effects of baicalein. Furthermore, the higher proportion of green-to-red cells indicate that baicalein can achieve the anti-virulent effects without bactericidal effects. The reduced total number of cells in the test group is attributed to the biofilm-inhibitory effect of baicalein and the consequent loss of free-floating cells, rather than bactericidal effects.

**Figure 7:**
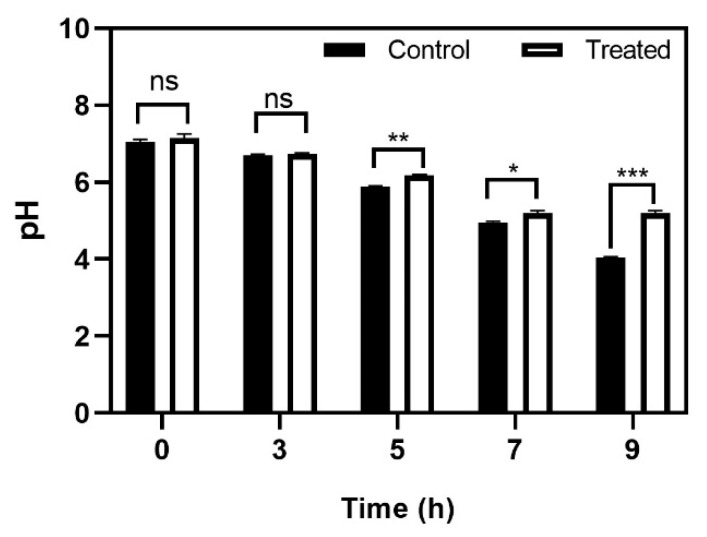
Effect of baicalein on *S. mutans* acid production. The time dependent effect on glycolytic pH drop. Student *t*-test was used for analysing significance and *p* < 0.05 was considered significant. * denotes *p* ≤ 0.05, ** denotes *p* ≤ 0.01 and *** denotes *p* ≤ 0.001.

Acid production serves two roles in the caries process. Its primary role is to maintain an acidic environment where the non-aciduric microbes cannot survive, contributing to dysbiosis. Secondly, acid production is also responsible for the acidic microenvironments within the biofilm, resulting in demineralization of the contacting tooth surface, leading to cavity formation [27,28]. Our results showed that baicalein did not allow the pH to drop below 5.2–5.5 which is considered the critical pH for enamel demineralization [29]. The pH of Baicalein solution in BHI media and supplemented with 20 mM sucrose was maintained at pH 7.4 ± 0.1 and in the sterile distilled water was maintained at pH 6.4 ± 0.1. Baicalein treated groups demonstrated a relative increase in pH from the 5th hour of incubation. The pH of ~5.2 was maintained until 9 h of treatment, while the pH dropped to ~4 for the control group (Figure 7). 

### 2.2. Baicalein Downregulates Several Genes That Regulate Metabolism, Biofilm, and Virulence

Gene expression studies showed that baicalein can negatively affect the expression of various interconnected virulent genes with multiple overlapping functions which are a part of the inherent *S. mutans* gene network. It is to be noted that there is a significant difference in the downregulation of certain genes between the mid-log phase biofilm cells and late-log phase biofilm cells. The spatial–temporal variations are naturally observed in the case of biofilm gene expression [30]. This was proven in the case of *E. coli* [31,32]. While the temporal variation in *S. mutans* biofilms is yet to be explored, it can be understood that the variations in the gene expression pattern in the biofilm cells are apparent. A similar trend is observed in our study where the downregulation is more in the mid-log phase as compared to the late-log phase. Due to the complexity of the biofilm cells at different developmental stages, the difference in the gene expression pattern is expected.

**Figure 8:**
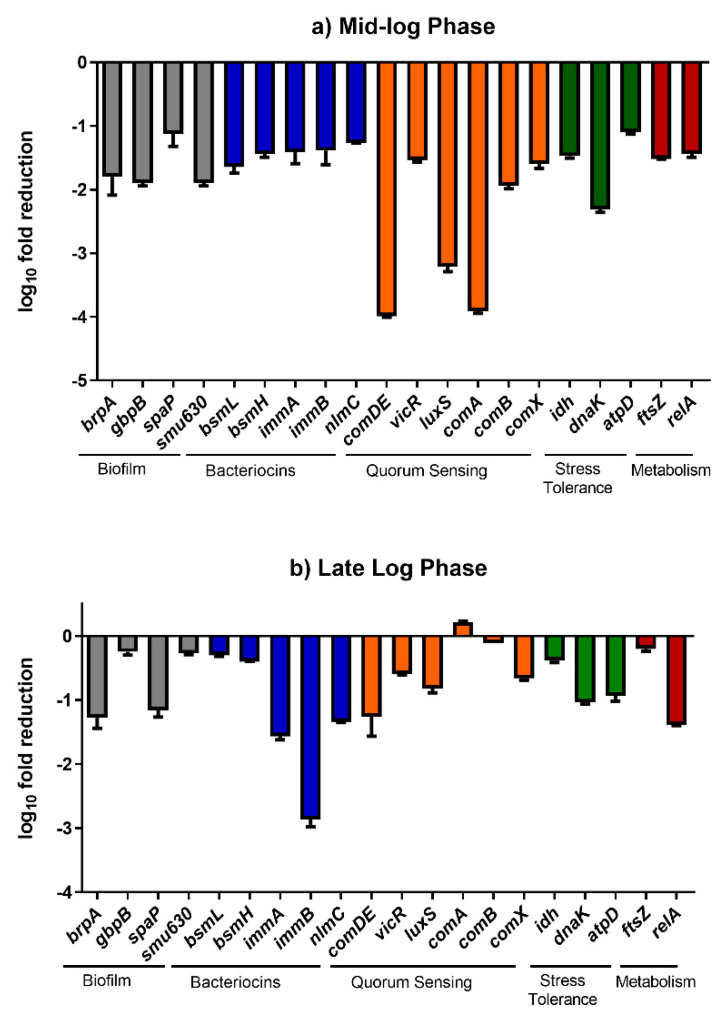
The transcriptional changes (fold reduction) in the expression of various genes in *S. mutans* biofilm cells on treatment with baicalein.

The time points for the qRT-PCR analysis were chosen based on the time during which the planktonic cell culture reaches it logarithmic phase (7th hour: Mid log phase and 9th hour: Late log phase). It should be noted that at 7th and 9th hour, there was a significant reduction in the cell-cell hydrophobicity and auto aggregation. Hence, we chose these two points to understand the temporal regulation in gene expression in treatment with baicalein. qRT-PCR analysis demonstrated that the effect of baicalein on downregulation of several genes on *S. mutans* biofilm cells was temporal, i.e., the downregulation at the 7th hour was relatively higher than that observed at the 9th hour (Figure 8). An approximate 4 log_10_ fold reduction of *comDE* expression was observed at the 7th hour, indicating a hindrance in the bacterial two-component system as well as competence development. Similarly, ~3 log_10_ fold reduction was observed in the expression of *luxS* which implies inhibition of quorum sensing and a reduction in cell competence. However, at the 9th hour after treatment, the fold reductions in expression reduced to approximately 1.4 log_10_ fold and 1.9 log_10_ fold for *comDE,* and *luxS,* respectively. Other quorum sensing genes such as *comA* and *comB* which displayed a 3.9 log_10_ fold and 2 log_10_ fold reduction respectively in the 7th displayed almost no change in expression in the 9th hour. Similarly, a relative downregulation of biofilm related genes *atlA, ftsz, vicR*, *gbpB* and *brpA* in the 7th hour, was decreased at the 9th hour. A similar trend was also observed in other virulence genes such as *idh*, *bsml, bsmH*, *comX* and *atpD*. By contrast, *brpA* and *spaP*, which are responsible for surface interaction and adhesion, and other virulence genes such as *dnaK*, *immA* and *relA* sustained their downregulation at both time points. Interestingly the fold reduction for *immB* increased from 1.4 log_10_ fold in the 7th hour of treatment to 2.8 log_10 fold_ in the 9th hour of treatment. 

A significant downregulation in the quorum sensing genes, including *luxS, comDE, comA* and *comB*, indicates that baicalein could significantly reduce the synchronized expression of virulence [33,34]. The *comDE, comA* and *comB* also play a role in conferring cell competence and eDNA in *S.mutans* is known for its role in biofilm formations and maturation [8]. Furthermore, the significant fold reduction observed in the genes crucial for biofilm formation further validated our findings from the biofilm inhibition assay. The downregulation of *brpA* might also responsible for causing a ripple effect in the expression of other virulence genes including *atlA* which is responsible for biofilm formation and *spaP* which plays a role in sucrose independent cell adhesion [4]. The reduction in the expression of dnaK sheds light on baicalein’s ability to hinder stress tolerance and recovery [35]. Genes, such as *immA* and *immB,* which are responsible for the secretion of bacteriocins also play a role in conferring immunity against antimicrobials including fluoride and chlorohexidine. The sustained downregulation of *immA* and a significant time dependent increase in downregulation of *immB* on treatment with baicalein showed that this compound could possibly synergise with conventional therapeutics [36]

### 2.3. Baicalein and Fluoride Synergistically Inhibit S. mutans Biofilm Formation

**Figure 9:**
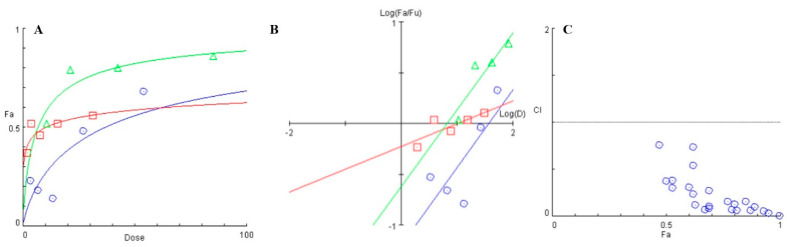
(**A**) Dose-response curve plotted between the fraction affected (fa) of biofilm formed and drug dose (μg/mL). (**B**) Median effect plot depicting the logarithmic transform of the ratio of the fraction affected (fa) and fraction unaffected (fu) as a function of the logarithmic transform of the dosage of individual treatment- (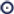) baicalein, (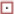) Fluoride and combination (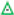) of Baicalein and Fluoride. (**C**) Combination Index (CI) versus Fraction affected (fa) plot for various combinations of baicalein and fluoride when tested for *S. mutans* biofilm inhibition (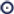) various combnations of baicalein and fluoride.

Phytochemicals, including flavonoids like baicalein are known inhibitors of energy metabolism, and studies have also proven that a synergistic effect can be obtained when administered in combination with conventional antimicrobials [37]. Fluoride treatment is currently used widely for as a means to prevent dental caries by inhibiting the demineralization of the tooth surface, and by enhancing remineralization [38]. However, the presence of biofilms prevents the adsorption of fluoride on to the tooth surface [39]. Having confirmed that baicalein can inhibit biofilm formation, we further asked if it could synergize with fluoride to inhibit biofilms. The Combination Index (CI) values were less than 1.0 for all combinations of sodium fluoride and baicalein showing synergy (Table S2). The dose-effect curve (Figure 9A) revealed a significant increase in the Fa (Fraction affected) values for various combinations of baicalein and fluoride when compared to individual drug treatment. Further, the median effect plot (Figure 9B) showed a significant increase in the combinatorial dose-response when compared to the dose-response of only fluoride. The left-skewed combinatorial curve points towards a more efficient drug response when compared to the treatment by only baicalein. The CI plot (Figure 9C) for the set for concentration combinations tested indicates synergy. 

## 3. Material and Methods

### 3.1. Bacterial Strains and Culture Conditions 

*S. mutans* UA159 (a well-characterized cariogenic pathogen and biofilm former) and *Streptococcus gordonii* ATCC35105 (an oral commensal) were grown in Brain Heart Infusion broth (BHIB; Himedia Laboratories Pvt. Ltd, Mumbai, India) supplemented with 20 mM sucrose. The cultures were maintained as glycerol stocks stored at −80 °C. For all assays, the inoculum was prepared by harvesting the cells until they reach the log phase, centrifuged and resuspended in fresh BHIB containing 20 mM sucrose and adjusted to OD_595_ 0.1 (~1.5 × 10^8^ CFU mL ^−1^). The bacterial cells were incubated anaerobically (5% CO_2_) at 37⁰C under static conditions [40]. All experiments were performed in triplicates on three independent occasions.

### 3.2. Test Compound Preparation

Baicalein (>98% purity) and Dimethyl sulfoxide (DMSO) was purchased from Tokyo Chemical Industry Co., Ltd (Japan). The stock solution was prepared by dissolving baicalein in DMSO. The working solution of baicalein was prepared by diluting it in BHIB to maintain the final DMSO concentration of 0.9% [23].

### 3.3. Effect on Planktonic Growth

The test for effect of baicalein on growth of *S. mutans* and *S. gordonii* was performed using 96 well microtiter plates (MTP) with two-fold serial dilution in BHIB supplemented with 20 mM sucrose resulting in concentrations ranging from 0.39µM to 400µM. The starting inoculum of the test strains (~1.5 × 10^8^ CFU/mL) were prepared as mentioned earlier and added to the respective wells. The culture plates were incubated at 37 °C for 24 h in 5% CO_2_. After 24 h, bacterial growth was calculated based on the OD_595_ values [40].

### 3.4. Effects on Biofilm Biomass

The biofilm inhibitory effect of baicalein was tested using 96 well microtiter plate method [41]. The 96 well MTP method and incubation conditions were concordant with the growth analysis assay. After incubation, the planktonic cells were removed by gently washing the wells three times with phosphate buffer saline (PBS). The biofilms were then air-dried and stained with 0.2% crystal violet solution. The excess stain was washed with PBS, dried, and the biofilm was de-stained with 33% acetic acid. The OD_595_ was measured and the concentration at which baicalein inhibited 50% biofilm (MBIC_50_) was calculated.

### 3.5. Bacterial Surface Hydrophobicity Assay and Bacterial Aggregation Assay

The cell surface hydrophobicity and bacterial autoaggregation assays were done as previously [42] with slight modifications. Briefly, *S. mutans* culture (OD_595_ = 0.5) was aliquoted in BHIB with or without 200 μM baicalein and incubated at 37 °C under anaerobic conditions. Then, 2 mL of bacterial suspension in triplicates were collected at three different log phases (early, mid, and late) and centrifuged at 5000× *g* at 4 °C for 5 min. Finally, the cells were washed twice and resuspended in PBS.

For the cell surface hydrophobicity assay, Xylene (20% *v*/*v*) was added to the PBS culture suspension obtained above, and vortexed vigorously to separate the aqueous phase, after which OD_595_ values was measured for the aqueous phase. The time-dependent decrease in absorbance was considered as a quantitative measure of the cell surface hydrophobicity index (HPBI). The HPBI was calculated with the following formula: HPBI = (A_0_-A)/A_0_ × 100, where A_0_ and A are the absorbance reading before and after extraction with xylene. A HPBI >70% was scored as hydrophobic [24,42].

To determine the bacterial aggregation, absorbance at A_595_ (A_0_) values for the cell suspension mentioned above were initially recorded, then the absorbance (A_t_) was subsequently measures following incubation for 2 hours. The extent of autoaggregation was calculated using the following formula: Aggregation % = (1 − (A_t_/A_0_)) × 100, where A_t_ represents the absorbance at time t = 5, 7 and 9 h. The aggregated cells of the baicalein-treated and untreated control groups were also visualized microscopically after crystal violet staining for qualitative analysis. 

### 3.6. Scanning Electron Microscopic Imaging

Baicalein-treated and untreated control biofilms were grown on glass slides (5 mm × 5 mm) in BHIB containing 20 mM sucrose for 24 h at 37 °C with 5% CO_2_. The samples were then fixed by immersing in 25% glutaraldehyde for 1 h and dehydrated with ascending concentrations of ethanol (40%, 60%, 80% and 100%). The slides were then gold sputter-coated, and the biofilm topography was analyzed under a Scanning Electron Microscope (VEGA3, TESCAN Analytics, Brno, Czech Republic) at 10 kV. The obtained images were analyzed using the ImageJ software v1.53a to determine the number of adhered bacterial cells. 

### 3.7. Confocal Laser Microscopic Imaging

To further elucidate the effect of sub-inhibitory concentration of baicalein on the cell viability and architecture of the biofilm, confocal laser microscopy was performed. Biofilms were grown on sterile coverslips in BHIB containing 200 µM baicalein supplemented with 20 mM sucrose. The untreated biofilm was also grown to serve as the control. The biofilms were incubated for 24 h with 5% CO_2_ at 37 °C. The slides were then washed with sterile PBS to remove the planktonic cells and the biofilm was stained using BacLight Bacterial Viability Kit (ThermoFisher Scientific, Waltham, USA). The biofilms were then observed using a confocal laser scanning microscope (Olympus FLUOVIEW, FV1000, Tokyo, Japan). Z-stacks were obtained from at least 5 regions and the obtained images were further analyzed using COMSTAT package in MATLAB R2017b [43]. Additionally, the cell count was enumerated using the ImageJ software.

### 3.8. Glycolytic pH Drop Assay

The effect of baicalein on acid accumulation in biofilms was determined using the glycolytic pH drop assay. *S. mutans* bacterial cells were harvested at the mid-exponential phase and washed with a salt solution comprising of 50 mM KCl and 1 mM MgCl_2_ and then resuspended in the same with or without 200 μM baicalein. The samples were adjusted to a pH of 7.2–7.4 using 0.2 M KOH and incubated in anaerobic conditions at 37 °C. The pH was recorded (Digital pH meter with pH electrode, Elico Ltd, Hyderabad, India) at three different time points (5, 7 and 9 h) [44]. The pH of Baicalein solution in BHI media and supplemented with 20 mM sucrose was maintained at pH 7.4 ± 0.1 and in the sterile distilled water was maintained at pH 6.4 ± 0.1.

### 3.9. Gene Expression Analysis

The influence of baicalein on the gene profiles of *S. mutans* biofilm cells was evaluated using Quantitative Real Time-Polymerase Chain Reaction (qRT-PCR). Biofilms were grown in 6 well plates in BHIB supplemented with 20 mM sucrose with or without 200 μM baicalein (MBIC_50_) and incubated at 37 °C in anaerobic conditions. At different time points (7th and 9th hour), the biofilm cells were harvested by gently scraping with a sterile scalpel in the presence of PBS and washed thrice with the same. Total RNA was extracted following the manufacturer’s guidelines of HiMedia RNA Extraction Kit (MB613). The integrity and purity of the extracted RNA were verified using standard agarose gel electrophoresis and NanoDrop (Thermo Scientific, Waltham, MA, USA) respectively. The total RNA was converted to cDNA using the iScript™ cDNA Synthesis Kit using the manufacturer-recommended protocol. Relative quantification of gene expression was performed with 16sRNA as the house-keeping gene, using the Fast-Real-Time PCR System (Applied Biosystems, Foster City, CA, USA) (Table S1). Table S1 lists the primer sequences used for this study. Fold changes in gene expression levels were calculated using the 2^−ΔΔCt^ method. The experiment was performed in quadruplicates at each time point and was repeated on three independent occasions [40]. 

### 3.10. Combinatorial Effects of Baicalein and Fluoride

The checkerboard assay was performed to assess the combinatorial effect of baicalein and fluoride on *S. mutans* biofilm formation. Bacterial cultures were inoculated in 96 well plates as described in previous experiments, with combinations of varying concentrations of baicalein and fluoride. The concentrations ranged from 12.5 to 200 μM for baicalein and from 0 to 31.25 ppm for fluoride. Untreated wells with only BHIB and sucrose served as controls. Biofilm quantification was done as described earlier and the resultant combined effect on the biofilm formation was evaluated using the CompuSyn software for the calculation of CI (Combinatorial Index) to determine the extent of synergy [45]. CI value of <1 indicates synergy, >1 indicates antagonism and 1 refers to additive interaction [46].

### 3.11. Statistical Analysis 

The results were expressed as mean ± SD. GraphPad Prism software version 6.05 (GraphPad Software Inc., San Diego, CA, USA) was used for performing the statistical analysis. Significance was checked with Dunnett *t*-test for multiple comparisons and paired Student’s *t*-test (*p* ≤ 0.05).

## 4. Conclusions

Our results provide the first experimental evidence to support the hypothesis that the biocompatible natural molecule baicalein can inhibit two high value targets of *S. mutans* for the development of caries, i.e., biofilm formation and acid production, without growth inhibition or inhibition of commensal bacteria such as *S. gordonii*. This indicates that baicalein has the potential to reverse dysbiosis in multi-species biofilms. This study highlighted the antibiofilm, anti-quorum sensing and anti-virulence potential of baicalein on *Streptococcus mutans*. Baicalein also synergized with fluoride to inhibit *S. mutans* biofilms, indicating its potential to be developed into anti-caries modalities. Novel delivery approaches are now being developed for further investigations using multi-species biofilm models on different substrates. 

## Data Availability

The data presented in this study are available on request from the corresponding author.

## Supplementary Materials

The following are available online at https://www.mdpi.com/2079-6382/10/2/215/s1, Table S1. List of Genes and Primer Sequences used for transcriptome analysis by qRT-PCR; Table S2. The CI values for individual combinations of Baicalein and Fluoride. For all the tested combinations CI < 1 indicating synergy. (CI = 1 indicates addictiveness while CI > 1 indicates antagonism).

## References

[1] Klein M.I., Hwang G., Santos P.H.S., Campanella O.H., Koo H. (2015). Streptococcus mutans-derived extracellular matrix in cariogenic oral biofilms. Front. Cell. Infect. Microbiol..

[2] Pitts N.B., Zero D.T., Marsh P.D., Ekstrand K., Weintraub J.A., Ramos-Gomez F., Tagami J., Twetman S., Tsakos G., Ismail A. (2017). Dental caries. Nat. Rev. Dis. Prim..

[3] Kim D., Barraza J.P., Arthur R.A., Hara A., Lewis K., Liu Y., Scisci E.L., Hajishengallis E., Whiteley M., Koo H. (2020). Spatial mapping of polymicrobial communities reveals a precise biogeography associated with human dental caries. Proc. Natl. Acad. Sci. USA.

[4] Shanmugam K., Sarveswari H.B., Udayashankar A., Swamy S.S., Pudipeddi A., Shanmugam T., Solomon A.P., Neelakantan P. (2020). Guardian genes ensuring subsistence of oral Streptococcus mutans. Crit. Rev. Microbiol..

[5] Thurnheer T., Belibasakis G.N. (2018). Effect of sodium fluoride on oral biofilm microbiota and enamel demineralization. Arch. Oral Biol..

[6] Hirzy J.W., Connett P., Xiang Q., Spittle B., Kennedy D., McDuffie J.E. (2018). Developmental Neurotoxicity of Fluoride: A Quantitative Risk Analysis Toward Establishing a Safe Dose for Children. Neurotoxins.

[7] Duangthip D., Fung M., Wong M., Chu C., Lo E. (2017). Adverse Effects of Silver Diamine Fluoride Treatment among Preschool Children. J. Dent. Res..

[8] Liao S., Klein M.I., Heim K.P., Fan Y., Bitoun J.P., Ahn S.-J., Burne R.A., Koo H., Brady L.J., Wen Z.T. (2014). *Streptococcus mutans* Extracellular DNA Is Upregulated during Growth in Biofilms, Actively Released via Membrane Vesicles, and Influenced by Components of the Protein Secretion Machinery. J. Bacteriol..

[9] Rukayadi Y., Kim K.-H., Hwang J.-K. (2008). In vitro anti-biofilm activity of macelignan isolated fromMyristica fragrans Houtt. against oral primary colonizer bacteria. Phytother. Res..

[10] Dashper S.G., Mitchell H.L., Cao K.-A.L., Carpenter L., Gussy M.G., Calache H., Gladman S.L., Bulach D.M., Hoffmann B., Catmull D.V. (2019). Temporal development of the oral microbiome and prediction of early childhood caries. Sci. Rep..

[11] Chen W., Jiang Q., Yan G., Yang D. (2020). The oral microbiome and salivary proteins influence caries in children aged 6 to 8 years. BMC Oral Health.

[12] Wang Y., Wang S., Wu C., Chen X., Duan Z., Xu Q., Jiang W., Xu L., Wang T., Su L. (2019). Oral Microbiome Alterations Associated with Early Childhood Caries Highlight the Importance of Carbohydrate Metabolic Activities. MSystems.

[13] Marsh P.D., Head D.A., Devine D.A. (2015). Ecological Approaches to Oral Biofilms: Control without Killing. Caries Res..

[14] De La Torre B.G., Albericio F. (2020). The Pharmaceutical Industry in 2019. An Analysis of FDA Drug Approvals from the Perspective of Molecules. Molecules.

[15] Gao Y., Snyder S.A., Smith J.N., Chen Y.C. (2016). Anticancer properties of baicalein: A review. Med. Chem. Res..

[16] Kumar M., Kasala E.R., Bodduluru L.N., Dahiya V., Lahkar M. (2016). Baicalein protects isoproterenol induced myocardial ischemic injury in male Wistar rats by mitigating oxidative stress and inflammation. Inflamm. Res..

[17] Liang S., Deng X., Lei L., Zheng Y., Ai J., Chen L., Xiong H., Mei Z., Cheng Y.-C., Ren Y. (2019). The Comparative Study of the Therapeutic Effects and Mechanism of Baicalin, Baicalein, and Their Combination on Ulcerative Colitis Rat. Front. Pharmacol..

[18] Liu H., Dong Y., Gao Y., Du Z., Wang Y., Cheng P., Chen A., Huang H. (2016). The Fascinating Effects of Baicalein on Cancer: A Review. Int. J. Mol. Sci..

[19] Lijian J., Luo W., Chung L.P., Leung P.C., Zhang C., Leung K.C.-F., Jin L. (2017). Nanoparticle-encapsulated baicalein markedly modulates pro-inflammatory response in gingival epithelial cells. Nanoscale.

[20] Song J., Zhang L., Xu Y., Yang D., Yang S., Zhang W., Wang J., Tian S., Yang S., Yuan T. (2021). The comprehensive study on the therapeutic effects of baicalein for the treatment of COVID-19 in vivo and in vitro. Biochem. Pharmacol..

[21] Chen Y., Liu T., Wang K., Hou C., Cai S., Huang Y., Du Z., Huang H., Kong J., Chen Y. (2016). Baicalein Inhibits *Staphylococcus aureus* Biofilm Formation and the Quorum Sensing System In Vitro. PLoS ONE.

[22] Peng L.-Y., Yuan M., Wu Z.-M., Song K., Zhang C.-L., An Q., Xia F., Yu J.-L., Yi P.-F., Fu B.-D. (2019). Anti-bacterial activity of baicalin against APEC through inhibition of quorum sensing and inflammatory responses. Sci. Rep..

[23] Cao Y., Dai B., Wang Y., Huang S., Xu Y., Cao Y., Gao P., Zhu Z., Jiang Y. (2008). In vitro activity of baicalein against *Candida albicans* biofilms. Int. J. Antimicrob. Agents.

[24] Hasan S., Danishuddin M., Khan A.U. (2015). Inhibitory effect of zingiber officinale towards *Streptococcus mutans* virulence and caries development: In vitro and in vivo studies. BMC Microbiol..

[25] Nyenje M.E., Green E., Ndip R.N. (2012). Biofilm Formation and Adherence Characteristics ofListeria ivanoviiStrains Isolated from Ready-to-Eat Foods in Alice, South Africa. Sci. World J..

[26] Stewart P.S. (2003). Diffusion in Biofilms. J. Bacteriol..

[27] Takahashi N., Nyvad B. (2010). The Role of Bacteria in the Caries Process: Ecological perspectives. J. Dent. Res..

[28] Lamont R.J., Egland P.G. (2015). Dental Caries. Molecular Medical Microbiology.

[29] Enam F., Mursalat M., Guha U., Aich N., Anik M.I., Nisha N.S., Esha A.A., Khan M.S. (2017). Dental erosion potential of beverages and bottled drinking water in Bangladesh. Int. J. Food Prop..

[30] Beloin C., Ghigo J.-M. (2005). Finding gene-expression patterns in bacterial biofilms. Trends Microbiol..

[31] Besharova O., Suchanek V.M., Hartmann R., Drescher K., Sourjik V. (2016). Diversification of Gene Expression during Formation of Static Submerged Biofilms by Escherichia coli. Front. Microbiol..

[32] Domka J., Lee J., Bansal T., Wood T.K. (2007). Temporal gene-expression in *Escherichia coli* K-12 biofilms. Environ. Microbiol..

[33] Kaur G., Rajesh S., Princy S.A. (2015). Plausible Drug Targets in the *Streptococcus mutans* Quorum Sensing Pathways to Combat Dental Biofilms and Associated Risks. Indian J. Microbiol..

[34] Das T., Sharma P.K., Krom B.P., Van Der Mei H.C., Busscher H.J. (2011). Role of eDNA on the Adhesion Forces betweenStreptococcus mutansand Substratum Surfaces: Influence of Ionic Strength and Substratum Hydrophobicity. Langmuir.

[35] Jayaraman G.C., Burne R.A. (1995). DnaK expression in response to heat shock of Streptococcus mutans. FEMS Microbiol. Lett..

[36] Wang W.-L., Liu J., Huo Y.-B., Ling J.-Q. (2013). Bacteriocin immunity proteins play a role in quorum-sensing system regulated antimicrobial sensitivity of *Streptococcus mutans* UA159. Arch. Oral Biol..

[37] Lee Y.-S., Jung E.-K., Cha J.-D. (2014). Synergistic Effect between Baicalein and Antibiotics against Clinic Methicillin and Vancomycin-Resistant Staphylococcus aureus. Chemo Open Access.

[38] Neel E.A.A., Aljabo A., Strange A., Ibrahim S., Coathup M., Young A.M., Bozec L., Mudera V. (2016). Demineralization–remineralization dynamics in teeth and bone. Int. J. Nanomed..

[39] Watson P., Pontefract H., Devine D., Shore R., Nattress B., Kirkham J., Robinson C. (2005). Penetration of Fluoride into Natural Plaque Biofilms. J. Dent. Res..

[40] Balasubramanian A.R., Vasudevan S., Shanmugam K., Lévesque C.M., Solomon A.P., Neelakantan P. (2020). Combinatorial effects of trans -cinnamaldehyde with fluoride and chlorhexidine on Streptococcus mutans. J. Appl. Microbiol..

[41] O’Toole G.A. (2011). Microtiter Dish Biofilm Formation Assay. J. Vis. Exp..

[42] Ciandrini E., Campana R., Baffone W. (2017). Live and heat-killed *Lactobacillus* spp. interfere with *Streptococcus mutans* and Streptococcus oralis during biofilm development on titanium surface. Arch. Oral Biol..

[43] Heydorn A., Nielsen A.T., Hentzer M., Sternberg C., Givskov M., Ersbøll B.K., Molin S. (2000). Quantification of biofilm structures by the novel computer program comstat. Microbiology.

[44] Belli W.A., Marquis R.E. (1991). Adaptation of *Streptococcus mutans* and Enterococcus hirae to acid stress in continuous culture. Appl. Environ. Microbiol..

[45] Ruhil S., Kumar V., Balhara M., Malik M., Dhankhar S., Kumar M., Chhillar A.K. (2014). In vitro evaluation of combination of polyenes with EDTA against *Aspergillus* spp. by different methods (FICI and CI Model). J. Appl. Microbiol..

[46] Wientjes M.G. (2010). Comparison of methods for evaluating drug-drug interaction. Front. Biosci..

