# Supplementary material for: Baicalein Inhibits Streptococcus mutans Biofilms and Dental Caries-Related Virulence Phenotypes
Source: Antibiotics (Basel). 2021 Feb 21;10(2):215. doi: 10.3390/antibiotics10020215 (PMC7926557; doi:10.3390/antibiotics10020215)
Supplement: Supplementary file 1 [file antibiotics-10-00215-s001.pdf]

Supplementary File

Table 1. List of Genes and Primer Sequences used for transcriptome analysis by qRT-PCR.

S. no	Gene name	Gene function	Forward primer sequence	Reverse primer sequence	Reference
1	*ftsZ*	Cell division protein	CTGAGATGCCTGCTGCTGAA	GATTGCTGTGGCTCAGATGATG	[1]
2	*gyrA*	Encodes DNA gyrase subunit A	TTCGTACAAGTGTTGCCGATATCT	TCTAGGCGCATCACTTTGACA	[1]
3	*gbpB*	Glucan binding protein - B	ATGGCGGTTATGGACACGTT	TTTGGCCACCTTGAACACCT	[2]
4	*relA*	Virulence phenotypes	ACAAAAAGGGTATCGTCCGTACAT	AATCACGCTTGGTATTGCTAATTG	[2]
5	*gtfC*	Extracellular polysaccharide synthesis	GGTTTAACGTCAAAATTAGCTGTATTAGC	CTCAACCAACCGCCACTGTT	[2]
6	*atlA*	Autolysin like protein	GTTAGTTCTGGTTTTGACCGCAAT	CCCTCAACAACAACATCAAAGGT	[2]
7	*spaP*	Adherence to saliva-coated tooth	GACTTTGGTAATGGTTATGCATCAA	TTTGTATCAGCCGGATCAAGTG	[2]
8	*immB*	Bacteriocin immunity proteins	GCTAGAGAGGCAAATGCACA	CAGCAGCAGCTGAGAAGATG	[3]
10	*immA*	Bacteriocin immunity proteins	TCTCCCCTGCTTGTTCAGAT	GCTGGCAAATTCGCTTACTT	[3]
11	*bsmH*	Bacteriocin production	AGACATGTTAGCCGCTGTTGAAG	AAGCGCCTGTTCCAATCGTA	[3]
12	*bsmI*		GAAACAATGGATACAGAGACG	GGAACAATAAGAGGATTTGG	[3]
13	*atpD*	Essential for acid tolerance	CCAGGCGGTTCATTCATCTGAC	GGCGGGATTTCGGTATTTACTG	[4]
14	*ldh*	Reversible enzyme in tricarboxylic acid cycle	ACTTCACTTGATACTGCTCGTT	AACACCAGCTACATTGGCATGA	[5]
15	*dnaK*	Stress tolerance protein	GGTACAACAAACTCAGCAGTTGCAGTTCTT	CCCCATCTTAGATTTGATGGAAAGAATTGT	[6]
16	*recA*	Involved in DNA repair	GGATCCGAGAAAAAGATTGGCCAAAAGAAT	TAAAGACTCGGGCTTGGGACCTATTTTTAT	[7]
17	*brpA*	Cell division, stress tolerance, biofilm formation	GGAGGAGCTGCATCAGGATTC	AACTCCAGCACATCCAGCAAG	[2]
18	*comDE*	Competence histidine kinase and response regulator	ACAATTCCTTGAGTTCCATCCAAG	TGGTCTGCTGCCTGTTGC	[2]
19	*comB*	Competence development	CCAGTCCAAACCGTCAGACT	GCTGCTTTCCTTGTCTTTCG	[3]
20	*comA*		ACGAGCCTAACAAGGGGATT	CCCTGAGGCATTTGTTCAAT	[3]
21	*covR*	Regulation of EPS synthesizing enzymes	ACACGATTACAGCCTTTGATGG	CTTCTTAGCCACTTCAAGACC	[1]
22	*vicR*	Promotes biofilm architecture	TGACACGATTACAGCCTTTGATG	CGTCTAGTTCTGGTAACATTAAGTCCAATA	[2]
23	*comX*	Competence development	CTGTTTGTCAAGTGGCGGTA	GCATACTTTGCCTTCCCAAA	[3]
24	*luxS*	Autoinducer-2 synthesis	ACTGTTCCCCTTTTGGCTGTC	AACTTGCTTTGATGACTGTGGC	[8]

Table 2. The CI values for individual combinations of Baicalein and Fluoride. For all the tested combinations CI < 1 indicating synergy. (CI=1 indicates addictiveness while CI>1 indicates antagonism).

Baicalein Conc. (μM)	Fluoride Conc. (ppm)	Effect	CI	Interpretation
200.0	31.25	0.85	0.21	Synergistic
200.0	15.63	0.99	5.12E-6	Synergistic
200.0	7.81	0.93	0.09	Synergistic
200.0	3.9	0.89	0.16	Synergistic
200.0	1.95	0.96	0.05	Synergistic
100.0	31.25	0.63	0.57	Synergistic
100.0	15.63	0.81	0.16	Synergistic
100.0	7.81	0.87	0.095	Synergistic
100.0	3.9	0.69	0.32	Synergistic
100.0	1.95	0.77	0.20	Synergistic
50.0	31.25	0.62	0.40	Synergistic
50.0	15.63	0.8	0.08	Synergistic
50.0	7.81	0.79	0.09	Synergistic
50.0	3.9	0.62	0.24	Synergistic
50.0	1.95	0.47	0.72	Synergistic
25.0	31.25	0.63	0.25	Synergistic
25.0	15.63	0.69	0.1	Synergistic
25.0	7.81	0.69	0.09	Synergistic
25.0	3.9	0.53	0.32	Synergistic
25.0	1.95	0.49	0.38	Synergistic
12.5	15.63	0.6	0.20	Synergistic
12.5	7.81	0.68	0.05	Synergistic
12.5	3.9	0.53	0.24	Synergistic

References:

[1] S. Hasan, K. Singh, M. Danisuddin, P.K. Verma, A.U. Khan, Inhibition of Major Virulence Pathways of Streptococcus mutans by Quercitrin and Deoxynojirimycin: A Synergistic Approach of Infection Control, PLoS One. 9 (2014) e91736. https://doi.org/10.1371/journal.pone.0091736.

[2] M. Shemesh, A. Tam, D. Steinberg, Expression of biofilm-associated genes of Streptococcus mutans in response to glucose and sucrose, J. Med. Microbiol. 56 (2007) 1528–1535. doi:https://doi.org/10.1099/jmm.0.47146-0.

[3] G. Kaur, P. Balamurugan, S.A. Princy, Inhibition of the Quorum Sensing System (ComDE Pathway) by Aromatic 1,3-di-m-tolylurea (DMTU): Cariostatic Effect with Fluoride in Wistar Rats , Front. Cell. Infect. Microbiol. . 7 (2017) 313. https://www.frontiersin.org/article/10.3389/fcimb.2017.00313.

[4] V. Gabe, T. Kacergius, S. Abu-Lafi, P. Kalesinskas, M. Masalha, M. Falah, B. Abu-Farich, A. Melninkaitis, M. Zeidan, A. Rayan, Inhibitory Effects of Ethyl Gallate on Streptococcus mutans Biofilm Formation by Optical Profilometry and Gene Expression Analysis, Molecules. 24 (2019) 529. doi:10.3390/molecules24030529.

[5] Z. He, Z. Huang, W. Jiang, W. Zhou, Antimicrobial Activity of Cinnamaldehyde on Streptococcus mutans Biofilms , Front. Microbiol. . 10 (2019) 2241. https://www.frontiersin.org/article/10.3389/fmicb.2019.02241.

[6] Y. Matsumi, K. Fujita, Y. Takashima, K. Yanagida, Y. Morikawa, M. Matsumoto-Nakano, Contribution of glucan-binding protein A to firm and stable biofilm formation by Streptococcus mutans, Mol. Oral Microbiol. 30 (2015) 217–226. doi:10.1111/omi.12085.

[7] S. Inagaki, M. Matsumoto-Nakano, K. Fujita, K. Nagayama, J. Funao, T. Ooshima, Effects of recom-

binase A deficiency on biofilm formation by Streptococcus mutans, Oral Microbiol. Immunol. 24 (2009) 104–108. doi:10.1111/j.1399-302X.2008.00480.x.

[8] Z.T. Wen, D. Yates, S.-J. Ahn, R.A. Burne, Biofilm formation and virulence expression by Streptococcus mutans are altered when grown in dual-species model, BMC Microbiol. 10 (2010) 111. doi:10.1186/1471-2180-10-111.
